# Dr. John Ashurst, Jr.

**Published:** 1900-08

**Authors:** 


Dr. John Ashurst, Jr., of Philadelphia, Pa., died at his home, July 7, 1900, aged 61 years. His death was due to paralysis from which he had been afflicted for about two years. He was born in Philadelphia and graduated at the University of Pennsylvania in 1860. He served as a medical officer during the civil war. He was appointed professor of clinical surgery at his Alma Mater in 1877, and ten years later became professor of surgery. Dr. Ashurst was one of the best known American surgeons both as an author and teacher. He edited the International Encyclopedia of Surgery which appeared in 1881, besides contributing many valuable monographs to the literature of surgery.

